# Acute Kidney Injury Associated With Cholera

**DOI:** 10.7759/cureus.34101

**Published:** 2023-01-23

**Authors:** Anass Qasem, Syed Arman Rabbani

**Affiliations:** 1 Department of Nephrology, Ibrahim Bin Hamad Obaidallah Hospital, Ras Al Khaimah, ARE; 2 Department of Clinical Pharmacy and Pharmacology, RAK College of Pharmacy, RAK Medical and Health Sciences University, Ras Al Khaimah, ARE

**Keywords:** infectious disease, gastroenteritis, united arab emirates, acute kidney injury, cholera

## Abstract

*Cholera* is an acute infectious disease caused by Vibrio cholerae. Its clinical course varies from mild diarrhea to severe complications with hypokalemia, hyponatremia or hypernatremia, hypocalcemia, metabolic acidosis, and acute kidney injury. This is a case of a 20-year-old Asian man with recent travel history from Bangladesh who presented to the emergency department with abdominal pain and multiple episodes of watery diarrhea. He developed acute renal failure secondary to severe gastroenteritis, the cause of which was later confirmed to be cholera.

## Introduction

Cholera affects an estimated 1.3 to 4 million people worldwide each year, with 21,000 to 143,000 deaths [1]. Despite these numbers, there are sporadic reported cases of cholera in the United Arab Emirates [2]. Cholera is an acute infectious disease caused by ingesting food and water contaminated by Vibrio cholerae. Its clinical course varies from mild diarrhea to severe complications with hypokalemia, hyponatremia or hypernatremia, hypocalcemia, metabolic acidosis, and acute kidney injury [3,4]. Of these complications, untreated electrolyte imbalance, metabolic acidosis, and acute kidney injury can lead to death [5]. To prevent these complications, prompt and aggressive volume expansion by administration of intravenous fluids is recommended [6]. Few cases reports from different counties have been published highlighting acute kidney injury in cholera patients [7,8].

This is a case of a 20-year-old man with recent travel history from Bangladesh who presented to the emergency department with abdominal pain and multiple episodes of watery diarrhea. He developed acute renal failure secondary to severe gastroenteritis, the cause of which was later confirmed to be cholera.

## Case presentation

A 20-year-old Asian male presented to Ibrahim Bin Hamad Obaidallah Hospital, Ras Al Khaimah, United Arab Emirates, on 24th March 2022 with abdominal pain and multiple watery diarrhea episodes for the last 24 hours. The patient had nausea and multiple episodes of vomiting and denied blood in stools or any drug or antibiotic intake. He had no history of smoking or alcohol. However, the patient recently traveled to Bangladesh. On examination, the patient was alert and oriented but looked dehydrated. His blood pressure was 85/56 mmHg with tachycardia. The lungs were clear, with an oxygen saturation of 98% and a respiratory rate of 17 breaths/minute. The abdomen was soft and distended with normal bowel sounds.

**Figure 1:**
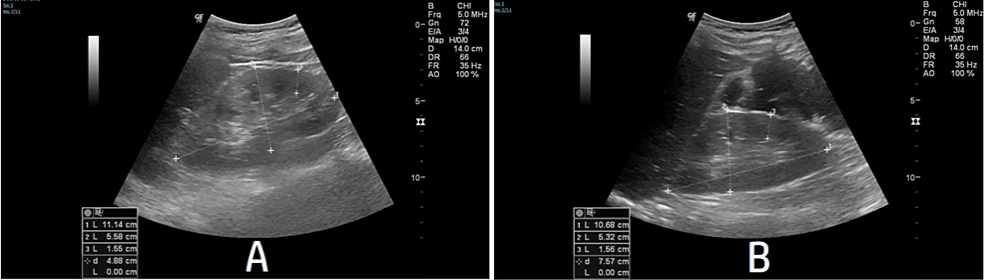
Ultrasound Images: A. Left kidney B. Right kidney

Initial laboratory investigations revealed neutrophilic leukocytosis (neutrophil count 40 x 10^3^/mcL), hemoconcentration (hemoglobin 18.8 g/dL, hematocrit level 54.3%), metabolic acidosis (pH of 7.1), low bicarbonate (8.9 mmol/L), hyponatremia (sodium 124 mmol/L), hypokalemia (potassium 2.9 mmol/L), elevated serum creatinine (438 µmol/L) and urea (16.2 mmol/L), reduced eGFR (15.6 mL/min), and rhabdomyolysis (creatinine kinase 6,094 IU/L, alanine transaminase (ALT) 83 IU/L and aspartate aminotransferase (AST) 153 IU/L). Furthermore, other biomarkers, C - reactive protein (174.5 mg/L) and procalcitonin (20.5 µg/L), were elevated. Urinalysis revealed proteinuria (urine protein of 4+) and hematuria (red blood cells of 8-10/high power field and hemoglobin of 3+). Chest radiograph and ultrasound abdomen were unremarkable (Figure 1). Ultrasound revealed that both left and right kidneys were of normal shapes and sizes with no calculi or back-pressure changes. 

Aggressive intravenous hydration was initiated with normal saline along with potassium chloride. Sodium chloride 0.9% was given at a variable rate according to the patient's response. Intravenous fluid rates ranged from 500 ml/h at first presentation with severe dehydration status to 83 ml/h during recovery. Intravenous potassium chloride supplementation was added to sodium chloride to correct mild hypokalemia. Additionally, the patient was given ceftriaxone and doxycycline. A urinary catheter was inserted, but no urine output was obtained. Renal function deteriorated further with worsening azotemia and persistent metabolic acidosis. Pre-renal acute kidney injury secondary to severe volume depletion was suspected. On day 3 of admission (26th March 2022), a femoral catheter was inserted, and the patient was initiated on intermittent hemodialysis with no ultrafiltration using K2 dialysate fluid and a dialyzer size of 1.8 m^2^.

Consequently, the patient received five dialysis sessions, and laboratory investigations showed a decrease in serum creatinine and urea levels with normal electrolytes. The patient was stable after each session of hemodialysis. In the meantime, stool culture was positive for Vibrio cholerae on 28th March 2022. On 9th April 2022, after 17 days of hospitalization, the patient's symptoms were entirely resolved, and he was discharged.

## Discussion

Cholera, if untreated, can become life-threatening with severe complications like refractory shock, acute kidney injury, coma, and death. Our patient had complicated cholera with severe electrolyte imbalance and acute kidney injury. He presented neutrophilic leukocytosis that may be attributed to the systemic inflammatory response to Vibrio cholerae infection. In addition, the patient had high hemoglobin and hematocrit levels, which is attributed to hemoconcentration secondary to diarrheal volume depletion. 

Furthermore, cholera is associated with several biochemical and acid-base abnormalities, including low serum potassium levels, low sodium, low chloride levels, and metabolic acidosis with a high anion gap [9]. Consistent with these abnormalities, our patient also presented with metabolic acidosis, low bicarbonate, hypokalemia, hyponatremia, and hypocalcemia.

Previous studies have reported that renal complications can develop during cholera as well as other severe gastrointestinal infections, as they did in our case [10]. Acute oligoanuric kidney injury could develop due to Vibrio cholerae infection itself or secondary to diarrheal volume depletion [10]. World Health Organization (WHO) recommends using intravenous fluids and antibiotics to manage cholera patients with severe dehydration [1]. Additionally, patients presenting with severe acute kidney injury not responding to conservative treatment may require renal replacement therapies [11]. In line with these recommendations, aggressive intravenous hydration, antibiotics, and hemodialysis were done subsequently, to which our patient showed complete resolution of his symptoms.

## Conclusions

In conclusion, we report an imported case of complicated cholera with severe electrolyte imbalance and acute kidney injury. The acute kidney injury in our patient may have been a result of renal ischemia secondary to dehydration leading to acute tubular necrosis. The patient was successfully managed by aggressive intravenous hydration and antibiotics, followed by hemodialysis.
